# Unusual Electromagnetic Signatures of European North Atlantic Winter Thunderstorms

**DOI:** 10.1038/s41598-017-13849-4

**Published:** 2017-10-24

**Authors:** Ondřej Santolík, Ivana Kolmašová

**Affiliations:** 1 Department of Space Physics, Institute of Atmospheric Physics, The Czech Academy of Sciences, Prague, Czechia; 2 Faculty of Mathematics and Physics, Charles University, Prague, Czechia; 3 Department of Upper Atmosphere, Institute of Atmospheric Physics, The Czech Academy of Sciences, Prague, Czechia

## Abstract

All lightning strokes generate electromagnetic pulses –atmospherics– which can travel over distances of thousands of kilometers. Night-side atmospherics show typical frequency dispersion signatures caused by sub-ionospheric propagation. Their analysis can be used to determine the distance to the source lightning, and therefore it represents a safe tool for investigation of distant thunderstorms, as well as for indirect observations of the lower ionosphere. However, such analysis has never been done on the dayside. Here we present the first results which show unusual daytime atmospherics with dispersion signatures originating from strong thunderstorms which occurred during winter months 2015 in the North Atlantic region. Using newly developed analysis techniques for 3-component electromagnetic measurements we are able to determine the source azimuth and to attribute these rare atmospherics to both positive and negative lightning strokes in northern Europe. We consistently find unusually large heights of the reflective ionospheric layer which are probably linked to low fluxes of solar X rays and which make the dayside subionospheric propagation possible. Although the atmospherics are linearly polarized, their dispersed parts exhibit left handed polarization, consistent with the anticipated continuous escape of the right-hand polarized power to the outer space in the form of whistlers.

## Introduction

It is known from the dawn of radio science^1^ that lightning strokes excite electromagnetic pulses which are now routinely used to localize lightning by triangulation techniques based on networks of radio receivers^2,3^. In the very low frequency (VLF) range below 30 kHz these electromagnetic pulses, called atmospherics, can propagate over thousands of kilometers from their source lightning. They travel inside the Earth-ionosphere waveguide which is formed by a conductive ground or sea on one side and by the lower edge of the ionospheric D-region on the other side^4,5^. Distances travelled by atmospherics are strongly dependent on the waveguide properties, primarily on the D-region reflectivity which exhibits significant diurnal, latitudinal, and seasonal variations. It also changes with varying solar activity, being prevalently controlled by the solar UV light and solar X-rays which ionize the neutral components in the D-region (N_2_, O_2_, Ar, CO_2_, He, O_3_, and H_2_O)^6^ during the day. The increased daytime ionization leads to a higher number of collisions with neutrals which result in low reflection heights, strong attenuation, and thereby short propagation paths of daytime atmospherics. VLF waves reflect from the lower ionosphere at an altitude of ∼60–70 km at midday but as high as ∼85–90 km at night^4,7,8^.

**Figure 1:**
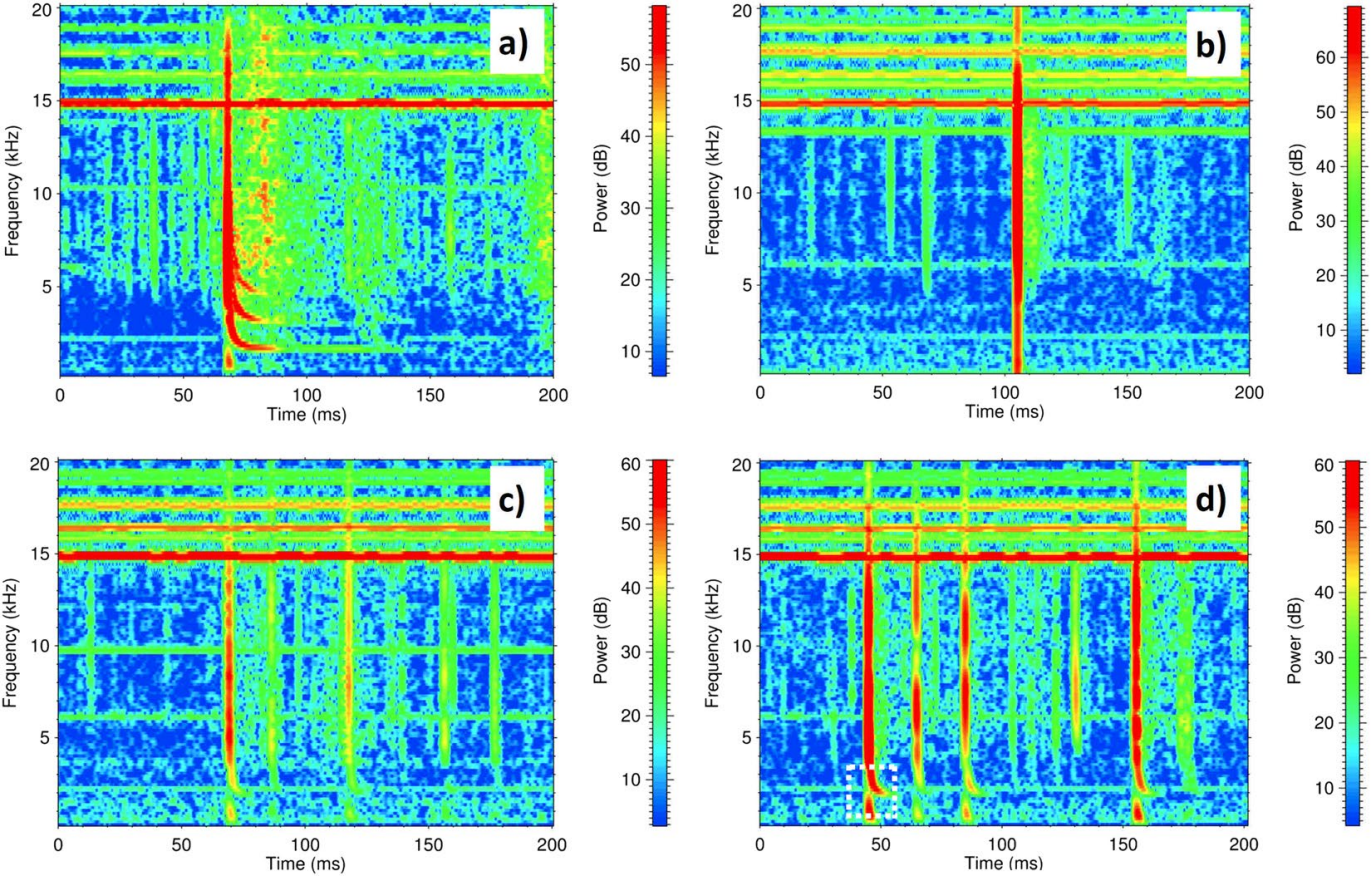
Examples of atmospherics. (**a**) Frequency-time spectrogram of the N-S magnetic-field shows a night-time tweek recorded on 12 Jan 2015 at 22:14:17.6 UTC. (**b**) Usual daytime atmospherics recorded on 14 Jan 2015 at 10:20:34.7. (**c**) Daytime atmospheric showing signs of dispersion recorded on 9 Jan 2015 at 12:36:48.2. (**d**) Unusual daytime tweek which is pronounced enough to be suitable for further analysis (9 Jan 2015 at 11:31:41.6; marked by a white dashed rectangle).

According to the waveguide theory^9^ an impulsive signal (in this case generated by a lightning discharge) excites different transmission modes. A zero-order mode travels in the waveguide at all frequencies but higher-order modes cannot propagate below their critical frequencies where their respective group velocities tend to zero. An atmospheric is then composed from these different waveguide modes. During the nighttime, many atmospherics exhibit a strong dispersion close to the critical frequencies of waveguide modes thanks to low attenuation and long propagation paths of electromagnetic VLF waves. These atmospherics are known as “tweeks” because of their typical sound when heard in a loudspeaker^1,10^. Figure 1a shows an example with visible dispersion close to three critical frequencies.

Tweeks were believed to be observed only during the local night or during the solar eclipse^11–13^ and their observation below a highly ionized daytime ionosphere has been thought impossible. Indeed, typical daytime observations show atmospherics without tweek signatures (Fig. 1b). Recent papers which report observations of tweeks in Antarctica during the sunrise and sunset^14^ and a detection of daytime tweek signatures originating in Japanese winter storms^15^, indicate that there might be specific and unusual state of the daytime ionosphere or specific types of thunderstorms, which might lead to the formation of daytime tweek signatures. This brought us to the idea to search for possible unusual electromagnetic traces of European winter thunderstorms using newly developed analysis techniques of our 3-component VLF measurements conducted in Southern France, sufficiently far from the North Atlantic region for possible tweeks to be observed.

## Results

### Experimental setup

Our VLF receiving station is placed in a favorable electromagnetic environment of the external measurement site of the Laboratoire Souterrain à Bas Bruit on the summit of La Grande Montange (43.941 N, 5.484E) close to Rustrel, France. We are using two perpendicular magnetic loop antennas with effective surfaces of 48 m^2^ a 10-cm spherical electric antenna located 2 meters above the ground, to monitor the vertical upward electric field component and horizontal eastward and southward magnetic field components. All sensors are equipped with integrated preamplifiers. To digitize and store the data we use a prototype of a multicomponent electromagnetic wave analyzer we have built during the preparation of the Resonance spacecraft mission and which undergoes extensive testing using natural electromagnetic signals. Every five minutes we record a 144-s long 3-component waveform sampled at 50 kHz.

### Data analysis

We have chosen the period from 7^th^ to 15^th^ January 2015 for our study. At that time a sequence of severe winter thunderstorms hit Ireland, the UK, Norway, Denmark, Germany, and Poland. During this period we have selected VLF data which were recorded at least two hours after the sunrise and two hours before the sunset, so that we can be sure the signals were received under daytime conditions. This gave us 59–62 data records of 144 s for each day, obtained during approximately 5 daytime hours. We have chosen the strongest atmospheric in each data record and searched for the signatures of frequency dispersion close to the critical frequency of the first-order waveguide mode in their time-frequency spectrograms. We have found promising unusual signs of dispersion for some of these atmospherics (Fig. 1c) and we have even successfully observed atmospherics with clearly pronounced tweeks (Fig. 1d). Their daily numbers varied irregularly between 0 and 17 cases.

In 545 data records collected during 9 stormy days we have altogether found 42 daytime tweeks which were strong, long and isolated enough to be analyzed for the first time using the newly designed “instantaneous frequency” method based on the Hilbert transform. This technique not only provides us with estimates of reflection heights but it also gives us propagation distances of the observed daytime tweeks (see Methods). To measure their arrival azimuth we use a direction finding technique based on our 3-component measurement of electromagnetic fields (see Methods).

### Locations of source lightning strokes

**Figure 2:**
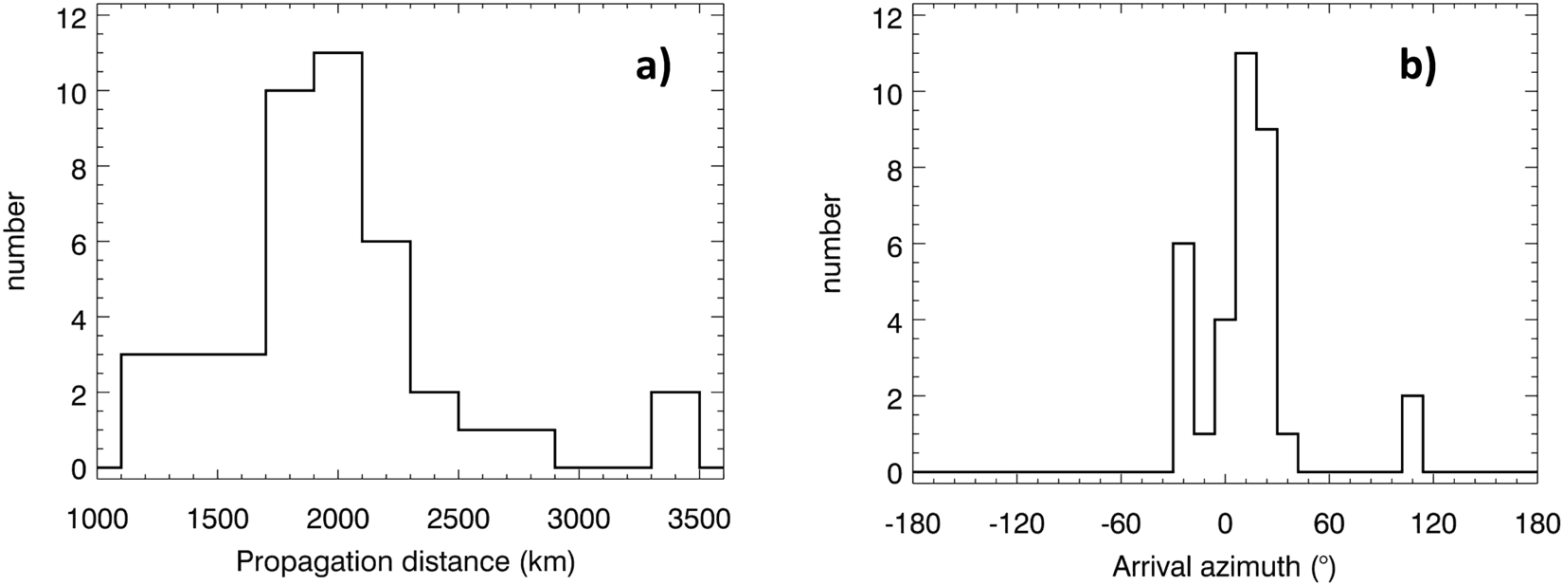
Distribution of propagation distance and azimuth of arrival of daytime tweek atmospherics. (**a**) Histogram of propagation distances *d* of the observed tweeks. (**b**) Histogram of the azimuth of arrival of the corresponding atmospherics.

For all 42 cases, the resulting propagation distances *d* (Methods, equation 2) of daytime tweeks vary from 1232 to 3379 km, with a peaked distribution around *d*~2000 km, as it is shown in Fig. 2a. From the least squares fits of the observed tweek traces we obtain an average experimental uncertainty of 19 km, while the maximum estimated uncertainties of the propagation distance induced by possible errors of the ground wave timing are around 60 km.

The estimated arrival azimuth fell into the interval between −43° and +36° in 40 cases (Fig. 2b) indicating that these tweek signals traveled to our receiver from the northern quadrant. The arrival azimuth was about 105° for the remaining two tweeks, pointing to the East. The experimental uncertainty of the arrival azimuth angles (calculated from the results obtained at different frequencies from 7 to 13 kHz) varies between 1.0° and 6.9° with an average value of 2.4°. For example, the event from Fig. 1d gives a propagation distance of 1291 ± 13.8 km and an azimuth of +20.4° ± 1.4°.

**Figure 3:**
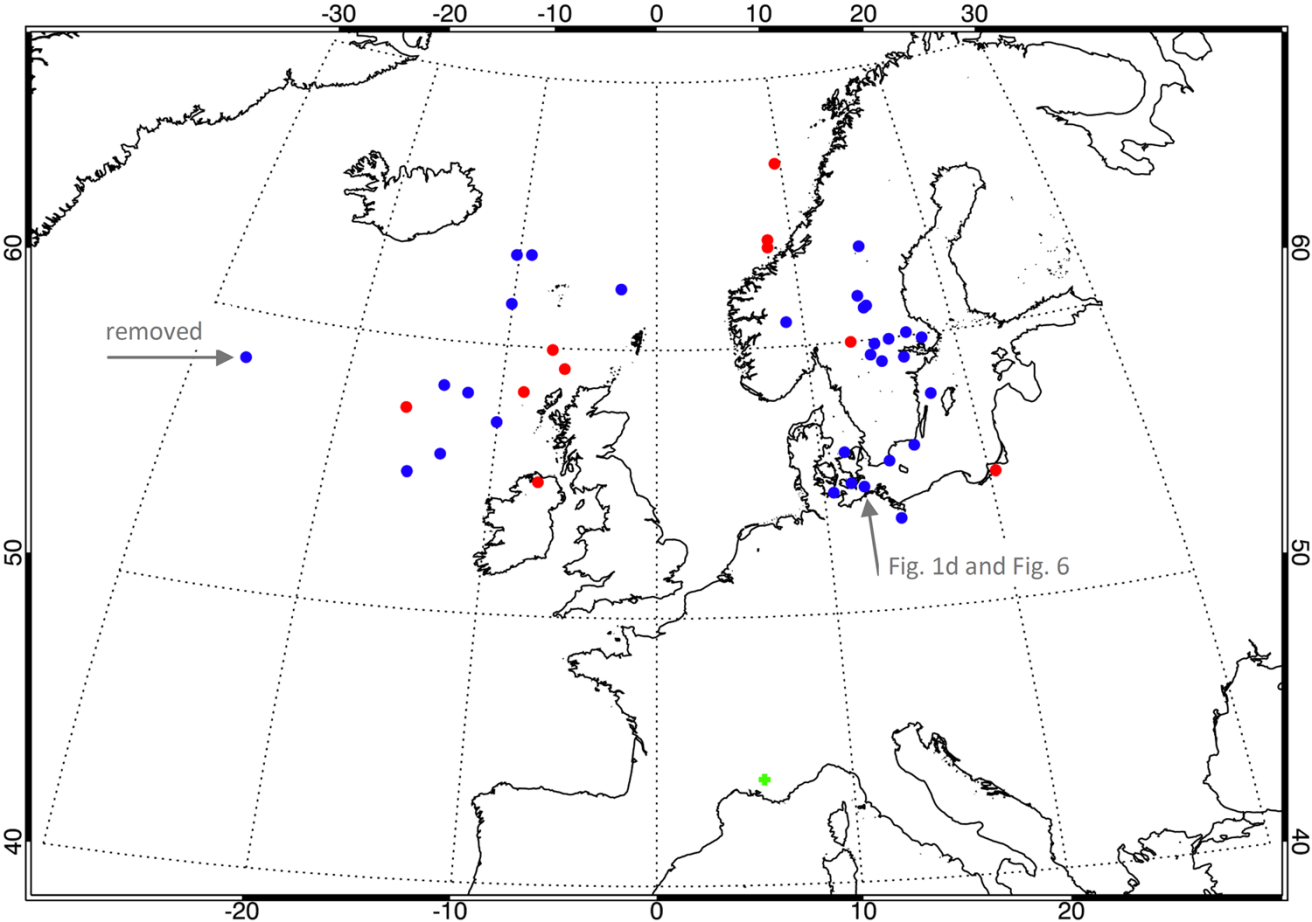
Locations of source lightning strokes. Red and blue dots represent positive and negative lightning strokes, respectively; a green cross shows the location of our VLF receiving station. (map has been plotted using the Interactive Data Language ver. 8.6.0). Horizontal arrow on the left marks an event which occurred 29 minutes before the local sunrise and was therefore excluded from the analysis. Another arrow highlights an event at a latitude of 54.6°N and a longitude of 12.4°E which is shown as an example in Figures 1d and 6.

Knowing the estimated propagation distances and arrival azimuths we can localize the source lightning strokes for the observed daytime tweeks. In 39 cases they were found in the region of the North-Atlantic thunderstorms (Fig. 3) and in 2 remaining cases the sources were found in an abnormally strong winter thunderstorm which brought snow and hail to the Middle East^16^ (not shown).

The polarity of ground wave peaks in the electric field waveforms (see the polarity estimation technique in Methods) indicates a positive polarity of 10 strokes (red dots in Fig. 3) and a negative polarity of 30 strokes (blue dots in Fig. 3) out of the total number of 40 source lightning strokes generating the observed tweeks and arriving from the northern quadrant. Additionally, we have found two positive source lightning strokes generating tweeks in the Middle East region. These results exceed usual fractions of positive lightning but our analysis is strongly influenced by a selection effect linked to our aim to analyze only strong cases exhibiting clear dayside tweek signatures.

**Figure 4:**
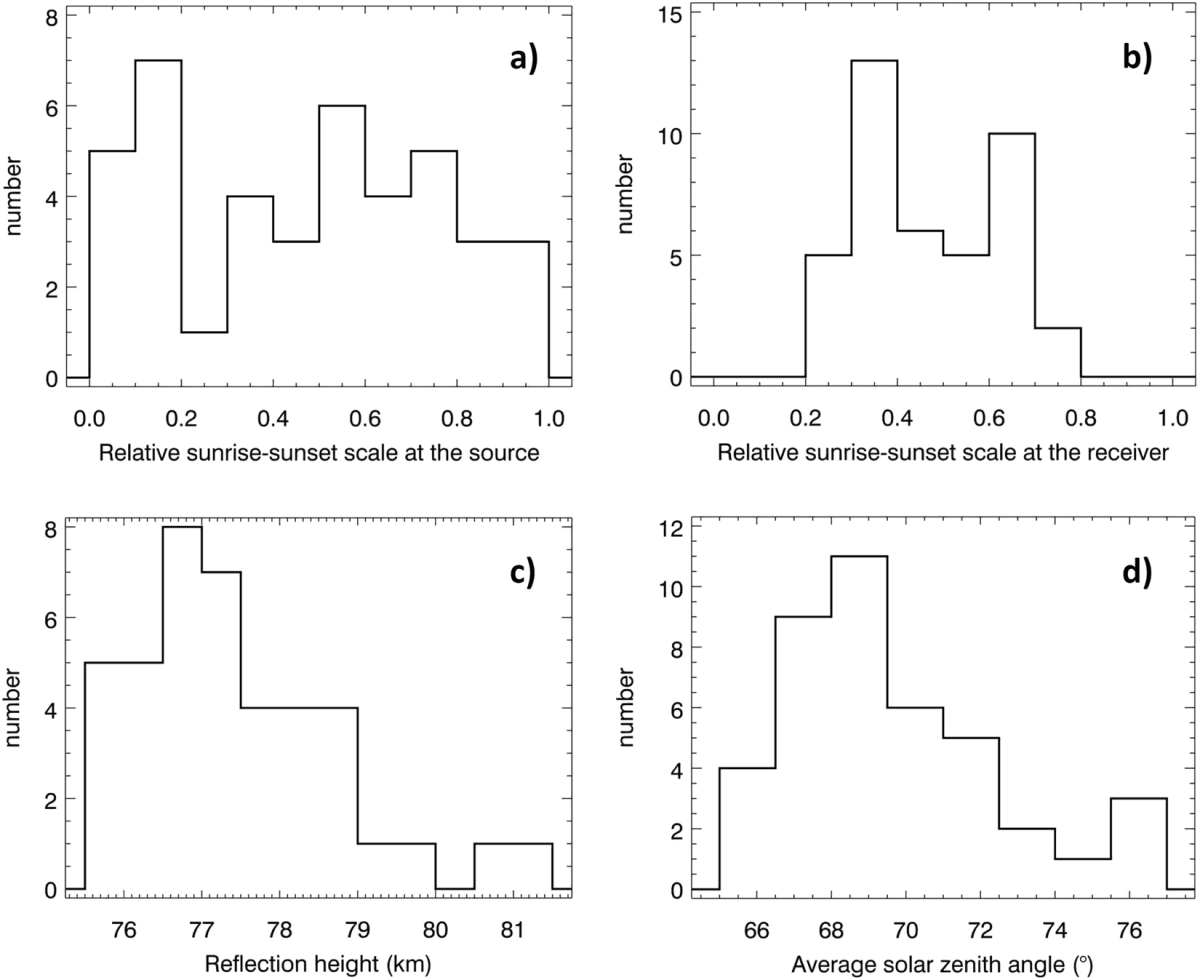
Distribution of propagation parameters of daytime tweek atmospherics. (**a**) Histogram of local times of the source lightning discharges on the scale of 0 (local sunrise) to 1 (local sunset). (**b**) Histogram of local times of reception of tweek atmospheric, on the same scale. (**c**) Histogram of reflection heights *h* (Methods, equation 3). (**d**) Histogram of the solar zenith angle averaged along the propagation path of each observed tweek atmospheric.

Only one of these preselected lightning sources (marked by a horizontal arrow on the left hand side of Fig. 3) occurred shortly before the local sunrise and was excluded from further analysis to ensure that the entire propagation path from the source to the receiver runs on the dayside for all the analyzed cases. All other sources correspond to lightning strokes which occurred on the sunlit part of the Earth. This is demonstrated by a broad distribution of local times of the source lightning discharges shown in Fig. 4a, while the distribution of the corresponding local times at the receiving site (Fig. 4b) is influenced by the choice of analyzing only the events which were recorded at least 2 hours after the sunrise and at least 2 hours before the sunset.

### State of the ionosphere

The critical frequencies *f*_*cn*_ (Methods, equation 2) have been transformed into reflection heights using equation 3. Their values for all the observed daytime tweeks vary from 75.56 to 81.06 km with an average experimental uncertainty of 76 m. The distribution of reflection heights obtained by this procedure is plotted in Fig. 4c. As the state of the dayside ionosphere necessarily depends on the solar zenith angle, we have calculated its average value along the propagation path of each observed unusual tweek atmospheric. Figure 4d shows that the Sun does not rise more than ~ 25° above the horizon in any of our cases, although the waves always propagate under the daytime conditions.

**Figure 5:**
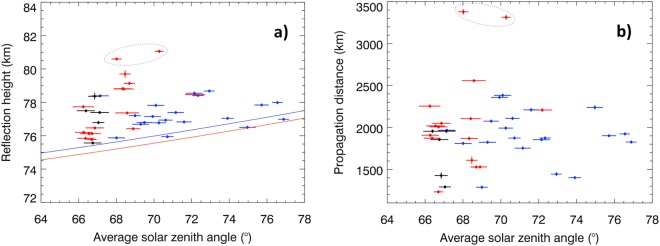
Relation of daytime tweek parameters to the solar zenith angle. (**a**) Reflection height of the observed tweeks as a function of the average solar zenith angle. Blue, red and black symbols respectively represent tweek signals propagating before, after and across the local noon. Error bars show standard deviations for reflection heights and solar zenith angle estimates. Blue and red solid lines show the model^7^ of morning (blue) and afternoon (red) reflection heights. (**b**) Propagation distance of the observed tweeks as a function of the average solar zenith angle with the same meaning of color. The outlaying pair of tweeks marked by grey ovals originates in the Middle Eastern thunderstorm.

Using this information we can compare our results with the model of reflection height as a function of the solar zenith angle, as it was obtained from measurements of amplitude and phase changes of VLF signals emitted by strong transmitters in the frequency range from 10 to 25 kHz ^7^. Figure 5 shows that the reflection heights of tweeks from our dataset were higher than the model in the majority of cases, even for high solar zenith angles which we mainly observe for the morning cases (in blue). However neither the experimentally obtained reflection heights (Fig. 5a), nor propagation distances (Fig. 5b) depend clearly on solar zenith angles within the limited range of values present in our dataset. Similarly, we do not observe clear differences of the reflection heights and propagation distances for the morning, noon, and afternoon cases, or for the positive and negative source lightning polarities (not shown).

### Polarization of dayside tweek atmospherics

**Figure 6:**
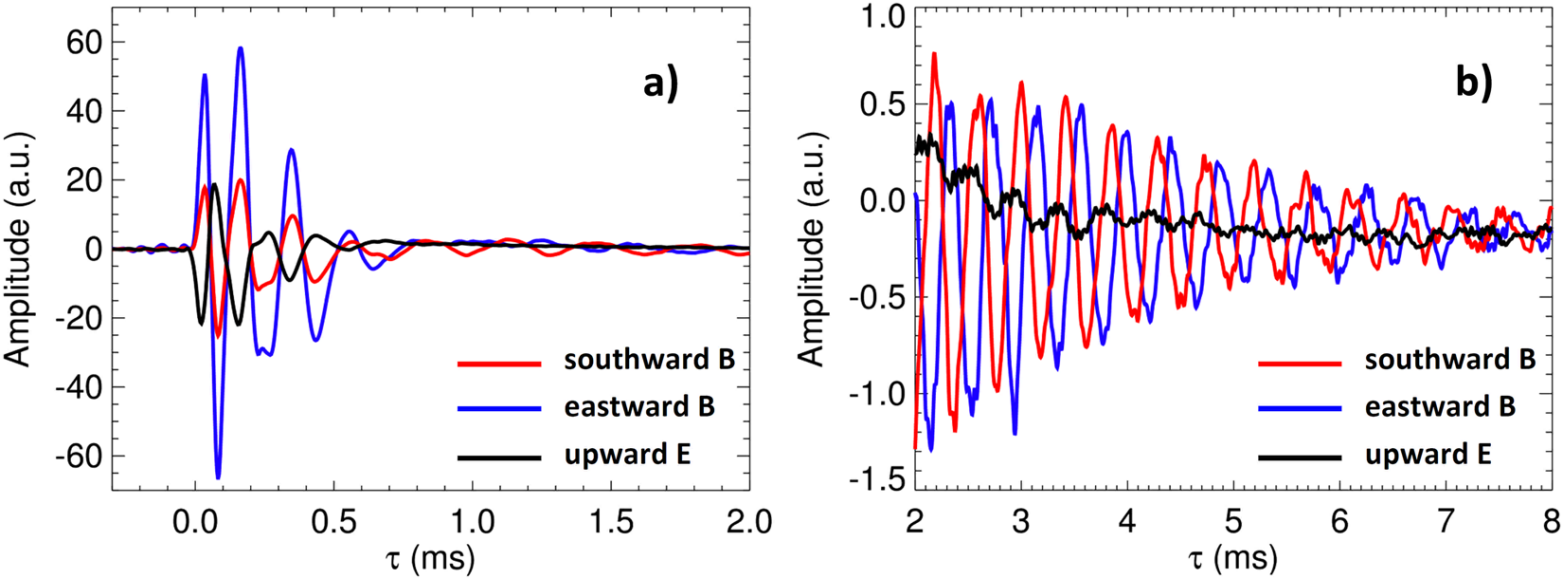
Typical waveforms of a daytime tweek atmospheric. The measurements have been collected on 9 January 2015 after 11:31:41.6 UTC. The southward and eastward magnetic field components, and the vertical electric component are represented by the red, blue, and black lines, respectively. Narrowband spectral lines caused by manmade interferences from VLF transmitters have been removed and the data were smoothed by a moving average filter to reduce the background noise. (**a**) Broadband linearly polarized atmospheric. (**b**) Immediately following quasi-sinusoidal circularly polarized signal of a tweek. Both figures are plotted versus the relative time τ (defined in Methods, below equation 2).

Using three components of the measured electric and magnetic field fluctuations we can examine the polarization properties of the observed electromagnetic waves. Figure 6 shows an example of received waveforms as a function of time relative to the arrival time of the ground wave signal. The time interval corresponds to a tweek atmospheric marked by a white dashed rectangle on the power spectrogram in Fig. 1d.

Figure 6a shows an initial intense negative peak of the measured vertical electric field, corresponding to a ground wave of a negative lightning discharge. At the same time, the horizontal eastward magnetic component shows an initial positive peak, and the southward magnetic component shows a smaller positive peak. The Poynting vector therefore must point to the south-southwest direction, in agreement with the estimated source position highlighted for this particular case in Fig. 3. For the ground wave and also for the following intense sky waves (ionospheric reflections) shown in Fig. 6a, the peaks of electric and both magnetic field components coincide. This confirms the expected linear polarization of the initial intense atmospheric.

At a relative time of 0.8–1 ms this picture starts to change in Fig. 6a: a much weaker quasi-sinusoidal tail of a tweek signature is formed close to the critical frequency of the first-order mode. This signature continues to be clearly visible in the two magnetic components in Fig. 6b. However, their waveforms are by 90° out of phase, with the southward component preceding the eastward one. This corresponds to a circular polarization with the same sense of rotation of the magnetic field vector as for the cyclotron motion of positive particles in the Earth’s magnetic field. This is conventionally labelled as the left-handed (L) polarization.

Very similar polarization pattern of linearly polarized initial ground wave and subsequent sky waves, followed by long quasi-sinusoidal tweek tails of left-hand circularly polarized waves are found for all the analyzed cases of daytime tweek atmospherics.

## Discussion

We have found that a thunderstorm occurring at higher latitudes of northern and north-western Europe during winter months was a source of tweeks, which were unexpectedly observed during the day. To our knowledge this is the first report showing propagation and polarization properties of daytime tweek atmospherics. It will be of great interest to follow this subject in the future as this unusual phenomenon is directly linked to a particular state of the ionosphere.

The obtained propagation distances of these daytime tweeks are 2–3 times shorter than tweek propagation paths which are typically observed during the night^12^ but their reflection heights are on average by ~5–10 km higher than heights typically obtained for standard daytime ionospheric conditions. Nevertheless they are still by ~10 km lower than typical reflection heights for the nighttime ionosphere^12^ and they are also by ~10–20 km lower than heights reported for Japanese winterstorms^15^.

Unexpected occurrence of daytime tweek atmospherics is consistent with a hypothesis that L-polarized waves are substantially less attenuated in comparison with the right-hand (R) polarized ones^17,18^. A question arises why the daytime ionosphere during north European winter was favorable for the observed L polarization to happen. It was shown that this polarization tends to occur when the anisotropy of the ionosphere becomes significant^18^. Then the L-polarized wave is reflected from the bottom of the ionosphere and the R-polarized wave escapes from the waveguide to the outer space as a whistler.

The ionospheric anisotropy is not significant for normal daytime conditions. To make it more significant, we need to increase the reflection heights and that is exactly what we observe in the analyzed cases. This increase of reflection heights leads to an unusually low daytime collision frequency and a sharper electron density profile^5^ resulting in an increased anisotropy. Winter conditions are favorable for this phenomenon to happen as the reflection heights tend to be larger for larger solar zenith angles^7^.

**Figure 7:**
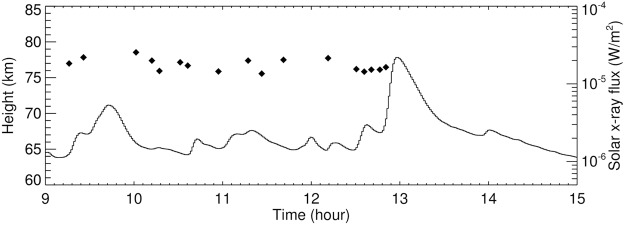
Soft X-ray flux coming from solar flares observed during the daytime tweek occurrence. The X‐ray data were recorded by Solar X-ray Imager onboard the Geostationary Operational Environmental Satellite 15 (GOES‐15) on 14 January 2015 between 9:00 and 15:00 UTC, and provided by the NOAA (National Oceanic and Atmospheric Administration) at http://satdat.ngdc.noaa.gov/sem/goes/data/new_full/. The solid line shows the solar flux at the wavelength from 0.1 to 0.8 nm. The diamonds show the occurrence of recorded daytime tweeks, including their reflection heights.

A remaining question is why the daytime tweek atmospherics haven’t been observed more often in winter thunderstorms. Here we need to discuss the variability of an important source of ionization of the ionospheric D region: X ray radiation linked to solar flares^8^. Figure 7 shows the soft X-ray flux measured by the Solar X-ray Imager onboard the GOES‐15 spacecraft on 14 January 2015, together with the times of observations of daytime tweeks. We abruptly stop to detect the tweeks with the arrival of a solar flare before 13:00 UTC which brought the largest observed soft X-ray flux during the analyzed interval of 7–15 January 2015. It therefore occurs that a major role, besides the winter conditions, probably plays the absence of intense solar X ray emissions which would otherwise increase the ionization of the dayside ionosphere.

## Methods

### Estimation of reflection heights and propagation distances of observed tweeks

Above the critical frequencies of the Earth-ionosphere waveguide, the group velocity *v*_*g*n_ of the electromagnetic wave propagating in the n^th^ waveguide mode can be written as a function of the critical frequency of the n^th^ mode *f*_*cn*_ and the frequency of the wave *f *^12^:

1 $${v}_{gn}=c\,\sqrt{1-{f}_{cn}^{2}/{f}^{2}},$$

where *c* is the speed of light. Assuming that waves at all frequencies are generated at the same time *t*_0_, they arrive to a propagation distance *d* at a time *t* = *t*_0_ + *d*/*v*_*gn*_. The expression for the frequency as a function of time then has two free parameters: the critical frequency *f*_*cn*_ and the propagation distance *d*:

2 $$f(\tau )=\frac{{f}_{cn}(c\tau +d)}{\sqrt{{(c\tau +d)}^{2}-{d}^{2}}},$$

where *τ* = *t* − *t*_0_ − *d* / *c* is time relative to the arrival time of the initial return stroke signal (ground wave) which we assume to propagate at the speed of light. The reflection height *h* can be then calculated from the critical frequency:

3 $$h=\frac{nc}{2{f}_{cn}}.$$

To analyze the behavior of the wave frequency of each observed tweek close to the critical frequency of the first waveguide mode we have selected one of the wave components which exhibited the most pronounced frequency dispersion in its time-frequency spectrogram. This always was the weaker of the two magnetic components: the southward magnetic component in 40 cases and the eastward magnetic component in 2 cases. The spectrograms of the electric field component did not show any signs of dispersion in our cases.

We have selected 10 milliseconds (500 samples) time interval which starts between 0.3 and 0.8 millisecond after the dominant peak of the corresponding atmospheric. This peak usually follows the ground wave signal by a small fraction of a millisecond. In this interval we have used a technique based on the analytic representation of the measured signal (previously implemented, for example, to analyze spacecraft data in the radiation belt region^19^). This representation is constructed in the frequency domain by suppressing the negative frequency components. In the time domain this is equivalent to adding an imaginary part to the real signal,

4 $$B(t)+{\rm{i}}\,{\rm{H}}\{B(t)\}=A(t)e{\rm{xp}}[{\rm{i}}\,\phi (t)],$$

where H{*B*(*t*)} denotes the Hilbert transform of the real signal *B*(*t*). *A*(*t*) is the instantaneous amplitude obtained as a modulus of the complex analytic signal, and the instantaneous frequency *f*(*t*) is obtained as the time derivative of the phase *φ*(*t*) of the analytical signal,

5 $$f(t)=\frac{d\phi (t)}{dt}.$$

**Figure 8:**
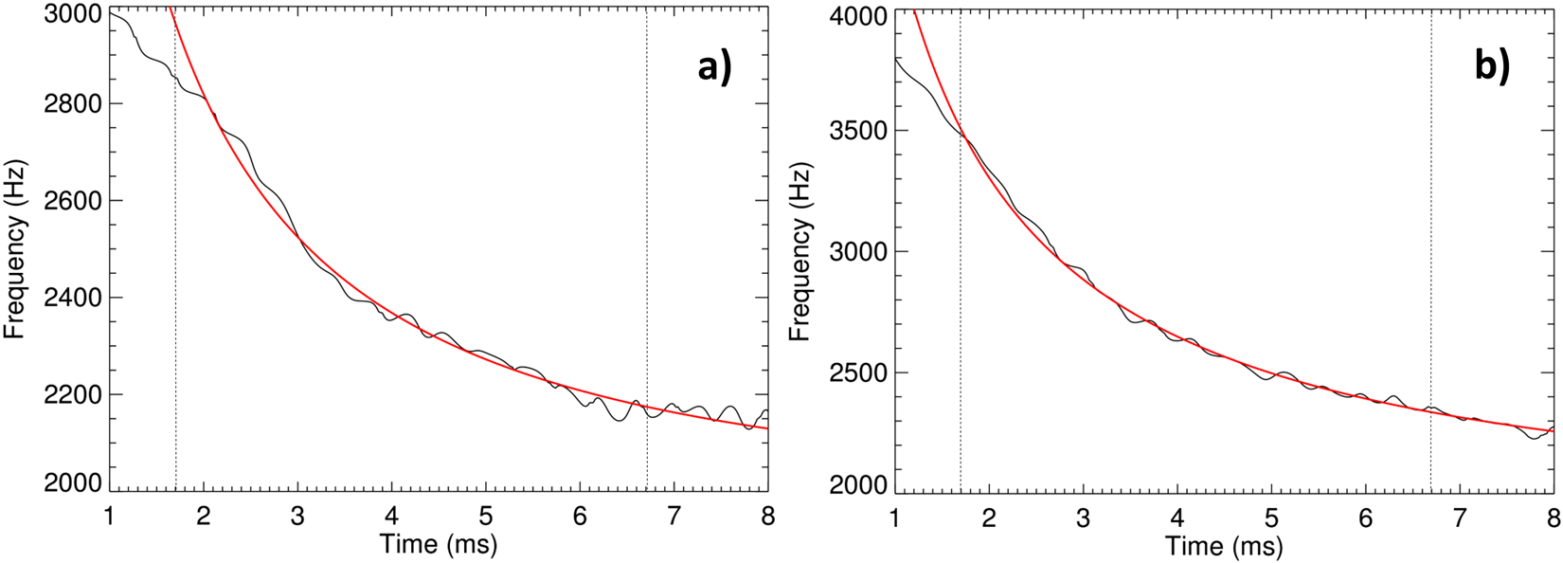
Examples of instantaneous frequency of tweek waveforms. Measured instantaneous frequency is plotted by black solid lines as a function of time relative to the arrival time of the initial return stroke signal; the nonlinear least squares fits are plotted by red lines. Black dotted lines delimit the time intervals in which the nonlinear least square procedure gave the best estimates of two free parameters (the first critical waveguide frequency and the propagation distance, equation 2). (**a**) Instantaneous frequency of a tweek recorded on 13 Jan 2015 at 13:35:06.1 UTC; the tweek signal travelled 1529 km in the Earth-ionosphere waveguide. (**b**) Instantaneous frequency of another tweek recorded on 13 Jan 2015 at 09:55:53.1; the tweek signal travelled 2723 km in the Earth-ionosphere waveguide.

The instantaneous frequency is plotted for two example cases in Fig. 8a,b, respectively (black lines).

We have used this estimated time evolution of instantaneous frequency as an input for a nonlinear least squares method to estimate two free parameters of the model in equation 2 (the critical frequency *f*_*cn*_ and the propagation distance *d*) and their standard deviations. As the investigated tweek signals were often polluted by other weaker atmospherics, we have been gradually reducing the original data set by 50 samples in order to achieve a lowest possible chi-square value and at the same time to keep the total number of samples above 200. The final numbers of samples in the individual time intervals for the least squares fit varied from 200 to 350. The resulting nonlinear fits for two differently distant events (1529 and 2723 km) are plotted by red solid lines in Fig. 8a,b.

### Arrival azimuths of the observed tweeks

To estimate the arrival azimuth of the selected tweek atmospherics we have used a direction finding technique based on the measurement of three components of electromagnetic fields at a single station. Our basic assumption is that the detected atmospherics are predominantly composed of the Transverse Magnetic (TM) waveguide modes. The TM waveguide waves are preferentially excited by vertical lightning currents^20^, their radiated electric field is oriented vertically, and the associated magnetic field is oriented horizontally and perpendicular to their propagation path. It means that our technique is based on an implicit assumption that the arrival azimuth of an atmospheric can be determined by measuring two horizontal magnetic field components and one vertical electric component of the incoming atmospheric. As its signal is very broadband, we use a predefined part of the frequency spectrum and we integrate the results over frequency.

As the first step the Fast Fourier Transform (FFT) is used for the selected time intervals of all three wave components sampled at 50 kHz to obtain a sum of sinusoidal complex signals with frequencies *f*_*k*_ ≤ 25 kHz, amplitudes *A*_*k*,*E*_, *A*_*k*,*S*_, and *A*_*k*,*V*_, and phases *φ*_*k*,*E*_, *φ*_*k*,*S*_, and *φ*_*k*,*V*_, respectively, for the eastward magnetic field component (*E*), southward magnetic field component (*S*), and vertical upward electric field component (*V*). For each frequency *f*_*k*_ we then calculate angle *α*_*k*_ using the amplitudes of both magnetic field components,

6 $${\alpha }_{k}={\rm{arctg}}(\frac{{A}_{k,E}}{{A}_{k,S}}).$$

Since the major part of the energy of atmospherics is concentrated in a several kHz wide frequency band around 10 kHz^21^ we have chosen the frequency interval from *f*_*k1*_ = 7 kHz to *f*_*k2*_ = 13 kHz in which we determine the wave arrival azimuth. We calculate the weighted average angle *α* from appropriate angles α_*k*_ with the respective weights *w*_*k*_ defined as the absolute magnitudes of the magnetic field in the horizontal plane,

7 $$\alpha =\frac{{\sum }_{k=k1}^{k2}{\alpha }_{k}{w}_{k}}{{\sum }_{k=k1}^{k2}{w}_{k}},$$

8 $${w}_{k}=\sqrt{{A}_{k,E}^{2}+{A}_{k,S}^{2}}.$$

As the amplitudes *A*_*k*,*E*_ and *A*_*k*,*S*_ are nonnegative; the values of *α* are contained between 0° and 90°. To obtain full arrival azimuths between −180° and +180°, we use the relative phases between all three components (*Φ*_*ES*_; *Φ*_*VE*_; *Φ*_*VS*_) of the investigated signals,

9 $${{\rm{\Phi }}}_{ES}=\arctan \frac{{\sum }_{k=k1}^{k2}{A}_{k,E}{A}_{k,S}\,\cos ({\phi }_{k,E}-{\phi }_{k,S})}{{\sum }_{k=k1}^{k2}{A}_{k,E}\,{A}_{k,S}\,\sin ({\phi }_{k,E}-{\phi }_{k,S})},$$

10 $${{\rm{\Phi }}}_{VE}=\arctan \,\frac{{\sum }_{k=k1}^{k2}{A}_{k,V}{A}_{k,E}\cos ({\phi }_{k,V}-{\phi }_{k,E})}{{\sum }_{k=k1}^{k2}{A}_{k,V}{A}_{k,E}\sin ({\phi }_{k,V}-{\phi }_{k,E})},$$

11 $${{\rm{\Phi }}}_{VS}=\arctan \,\frac{{\sum }_{k=k1}^{k2}{A}_{k,V}{A}_{k,S}\cos ({\phi }_{k,V}-{\phi }_{k,S})}{{\sum }_{k=k1}^{k2}{A}_{k,V}{A}_{k,S}\sin ({\phi }_{k,V}-{\phi }_{k,S})}.$$

For an isolated linearly polarized atmospheric unpolluted by any background noise or other interfering signals we would expect all components of the wave to have their relative phases equal to either 0° or ±180° in the whole range of frequencies. This assumption is not entirely satisfied in real conditions. Due to noisy background, interferences, propagation, and instrumental effects the resulting relative phases have deviated from their ideal values by 5–30°. Therefore, we round the relative phase values to the nearest multiple of 180° to retrieve the ideal values. This robust algorithm accompanied by the information about all polarization reversals of the signal inside our instrumentation was used to determine the correct quadrant of possible wave arrival azimuths and to estimate the azimuth of arrival of observed atmospherics.

### Polarity of source lightning strokes belonging to observed tweeks

Positive and negative lightning discharges are initiated in different thundercloud charge centers. The charge which is then being transferred in lightning channels during the return stroke phase is moving in opposite directions in cases of negative or positive lightning discharges^22^. Their polarity can be therefore recognized by the polarity of the initial ground wave pulses in waveforms of the vertical electric field component from atmospherics generated by these strokes.

As we are using a sign convention with a positive electric field pointing upward, we can expect negative ground wave pulses for negative strokes and positive ones for positive strokes^22^. However, the experimental determination of the polarity of ground wave pulses is sometimes difficult since the amplitudes of the sky wave pulses (reflected from the bottom of the ionosphere) start to be comparable with the ground wave amplitudes at distances above 500 km from the source lightning and they dominate the waveform recordings from distances above 1000 km. At distances larger than about 2000 km the ground wave might be already attenuated to the noise level^23^ in weak cases, and the first and the second sky wave (one or two reflections) become similarly strong. Nevertheless, as we only selected the strongest cases for this study, we believe that we were able to identify the polarity of ground waves for all 42 analyzed source lightning strokes which generated the observed tweeks. Most often, the strongest pulse in the sequence was found at the position of the first or the second sky wave pulse. The absolute amplitude of the weakest ground wave peak in our data set was still at 7% of the corresponding largest peak amplitude and well above the noise level.

### Data availability

The data used in this study are at http://bleska.ufa.cas.cz/lsbb/storage/elm/.
